# Objective and Measurable Biomarkers in Chronic Subjective Tinnitus

**DOI:** 10.3390/ijms22126619

**Published:** 2021-06-21

**Authors:** Dae-Woong Kang, Sung-Soo Kim, Dong-Choon Park, Sang-Hoon Kim, Seung-Geun Yeo

**Affiliations:** 1 Department of Otolaryngology, Head & Neck Surgery, School of Medicine, Kyung Hee University, Seoul 02447, Korea; kkang814@naver.com (D.-W.K.); hoon0700@naver.com (S.-H.K.); 2 Department of Biochemistry and Molecular Biology, School of Medicine, Kyung Hee University, Seoul 02447, Korea; sgskim@khu.ac.kr; 3 St. Vincent’s Hospital, The Catholic University of Korea, Suwon 16247, Korea; dcpark@catholic.ac.kr

**Keywords:** biomarker, tinnitus, pathophysiology

## Abstract

Tinnitus is associated with increased social costs and reduced quality of life through sleep disorders or psychological distress. The pathophysiology of chronic subjective tinnitus, which accounts for most tinnitus, has not been clearly elucidated. This is because chronic subjective tinnitus is difficult to evaluate objectively, and there are no objective markers that represent the diagnosis or therapeutic effect of tinnitus. Based on the results of studies on patients with chronic subjective tinnitus, objective and measurable biomarkers that help to identify the pathophysiology of tinnitus have been summarized. A total of 271 studies in PubMed, 303 in EMBASE, and 45 in Cochrane Library were found on biomarkers related to chronic subjective tinnitus published until April 2021. Duplicate articles, articles not written in English, review articles, case reports, and articles that did not match our topic were excluded. A total of 49 studies were included. Three specimens, including blood, saliva, and urine, and a total of 58 biomarkers were used as indicators for diagnosis, evaluation, prognosis, and therapeutic effectiveness of tinnitus. Biomarkers were classified into eight categories comprising metabolic, hemostatic, inflammatory, endocrine, immunological, neurologic, and oxidative parameters. Biomarkers can help in the diagnosis, measure the severity, predict prognosis, and treatment outcome of tinnitus.

## 1. Introduction

Tinnitus is derived from the Latin word “tinnire”, and is defined as a symptom of recognizing sound without external sound stimulation. Even in a quiet environment, approximately 94% of individuals feel tinnitus, but these sounds are not classified as clinically meaningful tinnitus; however, when the noise has a level of intensity that the patient feels distressing, it is considered meaningful tinnitus. Tinnitus is one of the most common symptoms in otology, along with hearing loss and dizziness, and appears as one of the symptoms associated with various clinical manifestations and several diseases. Tinnitus deteriorates the quality of life by inducing the problems, such as sleep disorders and psychiatric distress. In the UK, the social cost of tinnitus is reported to be approximately 750 million pounds per year [1,2,3,4].

Tinnitus can be classified into subjective and objective tinnitus. Objective tinnitus is a tinnitus that can also be heard by the examiner and originates from body organs, such as vascular and muscular, which can be classified into pulsatile tinnitus and non-pulsatile tinnitus. Pulsatile tinnitus is an arterial tinnitus that matches the heartbeat, whereas non-pulsatile tinnitus includes venous or muscular tinnitus that does not match the heartbeat. The most common causes of pulsatile tinnitus are vascular diseases, such as arteriovenous fistula, carotid artery stenosis, atherosclerosis, idiopathic intracranial hypertension, and venous hum, as well as muscle diseases, such as myoclonus [5,6]. Subjective tinnitus is the most common type of tinnitus in adults and is often associated with hearing loss. Depending on the initial trigger, it can be classified as primary tinnitus, which is associated with sensorineural hearing loss, or idiopathic and secondary tinnitus, which is related to organic causes [7].

Furthermore, it can be classified into central and peripheral types according to the problematic site in the auditory pathway [8]. Subjective tinnitus is known to be highly related to hearing loss, with sensorineural tinnitus accounting for approximately half and normal hearing tinnitus for more than 10% of cases [9]. Tinnitus can be classified into acute and chronic tinnitus according to the duration. Acute tinnitus is defined as such when the duration of tinnitus is less than six months, whereas chronic tinnitus is defined as a case that lasts more than six months. However, the exact criteria are not established and each study has individual definitions [7].

The mechanism of subjective tinnitus is not yet clear, and has been explained by several theories. First, as explained by the peripheral cochlear origin, damage to the cochlea and hearing loss are the causes of tinnitus, and is explained by the incongruity due to the difference in damage to the inner and outer hair cells or overactivity of neuroreceptors and neurotransmitters in the cochlea [10,11]. Second, as a change in the central nervous system, it is explained by the neurophysiological, neural synchrony, tonotopic reorganization, and neural plasticity theories, as the spontaneous firing of the central auditory system [12,13,14,15]. Of these, the well-known neurophysiological theory was proposed by Jastreboff, who suggested that not only the auditory system but also neurophysiology was associated with the occurrence of tinnitus [16]. According to this theory, because the acoustic information passes through the autonomic nervous and limbic systems, negative emotional reactions or discomfort are triggered, which leads to a vicious cycle of recognizing tinnitus even if there is no sound stimulus.

Most patients with tinnitus complain of chronic tinnitus that lasts for at least 3 to 6 months and is a subjective tinnitus with unknown cause. There are several studies on subjective chronic tinnitus, but the pathophysiology of tinnitus is not yet known. Although animal studies on tinnitus are actively being conducted, there are several limitations because of differences in their physiology from that of humans. To induce tinnitus in animals, short periods of ototoxic drug administration or acoustic trauma are used, but in humans, most tinnitus is chronic, and is mainly caused by chronic exposure over a long period of time. In addition, in humans, we can evaluate the characteristics of tinnitus through self-examination, but in animals, such measurements and evaluations are impossible and inaccurate. Therefore, although animal studies on the pathophysiology or treatment of tinnitus have been widely reported, they are rarely applied to humans [17]. Because of this unclear pathophysiology, the effect of tinnitus treatment currently being performed is unsatisfactory. According to a survey conducted in the United States among patients with tinnitus regarding tinnitus treatment, approximately 82.6% answered “not at all effectively” or “not very effectively”, and only 3.5% answered “very effectively” or “extremely effectively” [18]. As per a survey conducted in Sweden, about 46.5% of patients with tinnitus were not satisfied with the treatment or were not receiving treatment [19].

## 2. Aims

Although several studies have been conducted on tinnitus, one of the reasons for the difficulty in evaluating or treating tinnitus is the absence of objective markers that can indicate diagnosis, evaluation, and the effectiveness of treatment. In particular, since it is difficult to objectively evaluate chronic subjective tinnitus, most studies use audiology tests or self-questionnaire results as indicators. Thus, we made an attempt to find objective markers for the diagnosis or evaluation of the pathophysiology of tinnitus and to monitor the effectiveness of treatment.

A biomarker is defined as “a characteristic that can be objectively measured and evaluated as an indicator of normal biological processes, pathogenic processes or pharmacological responses to a therapeutic intervention” [20]. Various types of biological specimens were obtained to identify biomarkers for chronic subjective tinnitus. Although blood specimens require invasive sampling, they are relatively easy to obtain. Moreover, the concentrations of both individual and multiple biomarkers can be easily measured in blood samples. Urine specimens can be collected non-invasively during routine examinations, even in children. In addition, biomarkers of tinnitus are frequently assessed in saliva specimens, which have several advantages, including their easy and non-invasive collection, laboratory independence of sampling, and their usefulness in measuring basal levels of hormones. Despite these efforts, no study has reported the organization and classification of known biomarkers in chronic subjective tinnitus. Therefore, in this study, we categorized objective biomarkers related to diagnosis, prognosis, and the pathophysiology of tinnitus based on the results of published studies on chronic subjective tinnitus.

## 3. Review Methods

**Figure 1:**
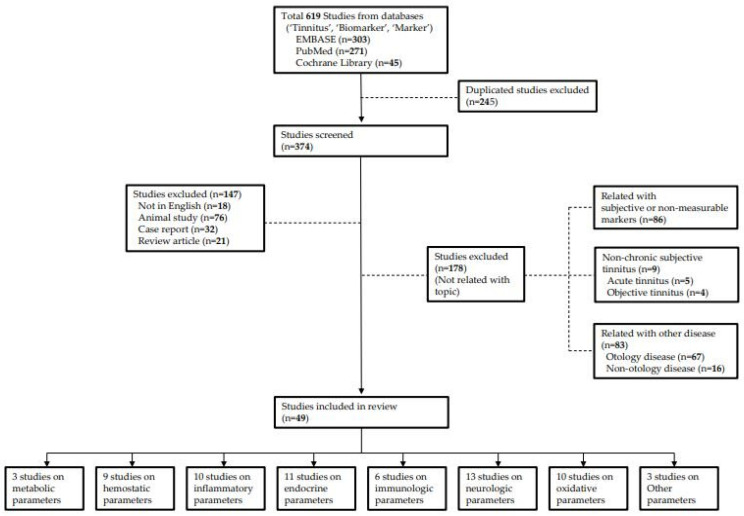
Flow diagram for review.

We investigated studies that reported biomarkers related to chronic subjective tinnitus published until April 2021. Data were collected by searching through the PubMed, EMBASE and the Cochrane Library databases using search terms “Tinnitus”, “Biomarker” and “Marker”. A total of 271 papers in PubMed, 303 papers in EMBASE, and 45 papers in Cochrane Library were found. Of these, duplicate studies, studies not written in English, review articles, and case reports were excluded. As mentioned above, because we defined biomarkers as indicators with physiological properties used in the evaluation and measurement of drug responses to normal biological processes, changes due to pathogenesis, and therapeutic interventions, studies that used subjective markers as indicators were excluded. In addition, because of the limitations of animal studies, animal studies and studies on acute tinnitus were excluded. Furthermore, studies focusing on other inner ear diseases (Meniere’s disease, neurodegenerative disease, vestibular schwannoma, or ototoxicity) and objective tinnitus were excluded. Finally, 49 studies were included in this study (Figure 1).

Since there is no standard categorization for tinnitus biomarkers so far, we classified the biomarkers into the following eight categories: (1) metabolic, (2) hemostatic, (3) inflammatory, (4) endocrine, (5) immunologic, (6) neurologic, and (7) oxidative parameters and (8) others. Considering the studies including parameters of various categories, out of 49 studies, three studies related to metabolic parameters, nine studies on hemostatic parameters, 10 studies on inflammatory parameters, 11 studies on endocrine parameters, six studies on immunologic parameters, 13 studies on neurologic parameters, 10 studies on oxidative parameters, and three studies on others were included.

## 4. Biomarkers in Chronic Subjective Tinnitus

### 4.1. Metabolic Parameters

Total cholesterol (TC)

Triglyceride (TRG or TG)

Low-density (LDL) and High-density lipoprotein (HDL)

Since the inner ear organ is an end organ that supplies blood flow to the stria vascularis or hair cells, it is sensitive to blood flow. The function of the end organ is impaired due to hypoxia caused by microcirculatory disturbance [21]. Dyslipidemia can lead to hypoxic damage in the inner ear by reducing blood flow to the end artery, and chronic hypoxia caused by this can affect the metabolism of the inner ear and cause tinnitus. Previous studies in guinea pigs and rats, reported that the inner ear function was reduced by a foreign body reaction caused by the accumulation of lipids in the inner ear, and the hearing function was preserved by lipid drug treatment [22,23]. In humans, reports have demonstrated the relationship between tinnitus and dyslipidemia. While there are reports that the severity of tinnitus was reduced through diet control in patients with tinnitus, some studies reported that the severity did not decrease even with administration of hyperlipidemia medications for more than four months in patients with subacute tinnitus [24,25,26]. However, the relationship between tinnitus and hyperlipidemia seems to be strong, as shown in studies using objective biomarkers. In a study that evaluated blood tests, intima media thickness (IMT) of the carotid artery, and the severity of tinnitus in patients with bilateral chronic subjective tinnitus with a prevalence of more than six months, Tinnitus handicapped index (THI) score, total cholesterol (TC), low-density lipoprotein (LDL), and hearing threshold were high in patients with tinnitus with high IMT [27]. In another study, tinnitus patients with normal hearing had lower mean blood HDL levels and higher TG levels than those of the control group [28]. In a study comparing patients with chronic subjective tinnitus, aged 18 to 70 years, with a control group to rule out the effects of aging, there was no difference in HDL levels between the two groups, but TC, TG, and LDL levels were high in the tinnitus group [29]. These results showed that changes in metabolic parameters, which are known to cause microcirculatory disturbance, increase the severity as well as the risk of tinnitus, similar to other inner ear diseases.

### 4.2. Hemostatic Parameters

Mean platelet volume (MPV)

Platelet distribution width (PDW), Platelet count (PC)

Plasma 11-dehydro-thromboxane B2 (11-dTxB2)

Platelets are one of the normal cells that exist in the blood and play an important role in blood hemostasis. In particular, the average size of platelets is represented by mean platelet volume (MPV), and the larger the average platelet size, the more vasoactive factors are involved; thus, hemostasis occurs well. MPV is related to cardiovascular or cerebrovascular diseases, such as diabetes mellitus (DM), hyperlipidemia, hypertension, and obesity [30,31]. Low MPV levels are also related to disease activity in inflammatory diseases, such as ulcerative colitis, Crohn’s disease, and chronic obstructive pulmonary disease, as they are involved in inflammatory reactions [32]. Despite this importance, the MPV value can be overlooked because it is relatively easily accessible. As mentioned earlier, considering the importance of blood flow in inner ear metabolism, the relationship between MPV and tinnitus should be carefully considered. In a study conducted to rule out the effects of age-related hearing loss (presbycusis) or sensorineural hearing loss in patients under 50 years of age with tinnitus and normal hearing, the average MPV level was higher in the tinnitus group than in the control group [33]. In other similar studies, although the sex and age of the two groups were different, the ratio of the patients with high MPV level, hypertension, DM, and hyperlipidemia was higher in the tinnitus group than in the control group [34]. Furthermore, MPV levels were related to the type of hearing loss in patients with tinnitus.

In a study in which tinnitus patients with hearing loss were divided into four groups according to the type of hearing loss (all frequency hearing—tinnitus [AFHL-TN], high-frequency hearing loss—tinnitus [HFHL-TN], non-hearing loss-tinnitus [NH-TN], and control), the average MPV level of the HFHL-TN group was slightly higher than that of the AFHL-TN group, but it was significantly higher than that of the NH-TN and control groups [35]. Some studies included platelet distribution width (PDW) and platelet count (PC), which are other values related to platelet activity. As expected, in one study, the MPV and PDW levels were higher in patients with tinnitus, but in another study, the levels of PDW and PC were higher, whereas the MPV levels were lower [36,37]. In a study on the relationship between the MPV level and the characteristics of tinnitus and hearing loss frequency, the MPV level was high in patients with tinnitus as expected, but there was no relationship between the high MPV levels and characteristics of tinnitus, such as intervals, sound pattern or duration, and hearing loss frequency [38]. As the relationship between tinnitus and platelet aggregation is known, studies have also been conducted on factors related to platelet activity. When platelets are activated, unstable thromboxane A2 differentiates into various substances. Among them, 11-dehydro-thromboxane B2 (11-dTxB2), which has a long half-life and is stable, plays a major role in vasoconstriction and platelet aggregation. It has already been reported that thromboxane levels play an important role in cardiovascular diseases in animal studies. In a study of tinnitus patients with normal hearing, no significant results were observed in prothrombin time (PT), international normalized ratio (INR), and fibrinogen, but 11-dTxB2 levels were higher in patients with chronic subjective tinnitus [39]. These results support the theory that the inner ear is vulnerable to microcirculation because of the lack of collateral vascular circulation, and hemostatic marker measurement is expected to help diagnose and treat tinnitus [40].

### 4.3. Inflammatory Parameters

Neutrophil/lymphocyte ratio (NLR)

Platelet/lymphocyte ratio (PLR)

CRP (C-reactive protein)

ESR (Erythrocyte sedimentation rate)

Tinnitus has a great influence on stress, and it is known that stress-related inflammatory reactions are important in the study of cardiovascular and other diseases [41]. The neutrophil/lymphocyte ratio (NLR) is the most commonly used marker of inflammatory response in patients with tinnitus to reveal the etiology of tinnitus. NLR is already used as a representative marker of the inflammatory response in cardiovascular and kidney diseases, and is known as a prognostic factor for facial palsy and sudden sensorineural hearing loss in otology [42,43,44]. In a previous study on tinnitus patients with normal hearing, the average NLR was higher in the tinnitus group than in the control group. In addition, when receiver operating characteristic (ROC) analysis was performed, the appropriate NLR cut-off value was 2.17 (sensitivity 28.6%, specificity 83.3%), and the risk of tinnitus increased by approximately 1.99 times when the NLR level was 2.17 or higher [34]. NLR levels have also been reported to be associated with the severity of tinnitus. In a study of patients with tinnitus for at least two weeks, the number of white blood cells (WBC), a traditional inflammatory marker, was not high in patients with tinnitus, but NLR was high in patients with severe tinnitus. In another study, NLR was positively correlated with the severity of tinnitus [45,46].

In addition, as an abnormality of the peripheral auditory system is considered as one of the causes of tinnitus, a study was conducted on audiologic assessment. For the auditory function test, not only pure tone audiometry (PTA) but also distortion product otoacoustic emission (DPOAE) is widely used to evaluate cochlear function in tinnitus patients with normal hearing. According to a study on DPOAE and tinnitus, the amplitude of DPOAE decreased by up to 93% in tinnitus patients with normal hearing [47]. Furthermore, the hearing threshold was increased at high frequencies in PTA in these patients [47]. A study has reported the relationship between DPOAE results and hearing loss frequency and hematologic test results in chronic subjective tinnitus. In this study, NLR was related to the mean bone pathway PTA value and discrimination value. According to the hearing loss frequency, the amplitude of DPOAE showed a negative correlation between the conductive hearing threshold at 4 kHz and 8 kHz, and the bone conductive hearing threshold at 4 kHz, and a positive correlation was shown at other high frequencies [48]. Similar studies related to tinnitus and high-frequency hearing loss have also been reported. In one study, the NLR of tinnitus patients with high-frequency hearing loss was higher than that of all-frequency hearing loss, tinnitus patients with normal hearing, or controls. PLR, an inflammatory marker used with NLR, was also reported to be higher in tinnitus patients with high-frequency hearing loss than in those with all frequency hearing loss [35]. However, some studies reported that NLR and PLR were not associated with tinnitus, and some even showed opposite results [37,40,49]. Significant results were not observed with respect to WBCs but ESR and CRP have shown elevated levels in several studies [36,37]. In conclusion, the above results suggest that the inflammatory response of the inner ear is partially involved in tinnitus, although there are no consistent results for some inflammatory markers. In particular, it presents the relationship between high-frequency hearing loss and tinnitus and provides evidence for the pathophysiology of tinnitus through inflammatory markers.

### 4.4. Endocrine Parameters

Serum cortisol

Adrenocorticotropic hormone (ACTH)

5-hydroxyindoleacetic acid (5-HIAA)

Salivary cortisol/cortisone

Plasma melatonin

As mentioned in the introduction, tinnitus not only interferes with the quality of life, such as daily life, study, and work, but may also result in serious psychiatric illness or even suicide [50]. This is because stimulation by tinnitus influences the auditory nervous system and the patient’s psychology through activation of the autonomic nervous system by the limbic system and cerebral cortex, based on the psychophysiological theory mentioned above. Studies related to stress-related hormones have been reported for the purpose of understanding the effects of tinnitus-induced stress on the endocrine system.

Under stressful conditions, the hypothalamic–pituitary–adrenal axis (HPA) is activated in the body, and cortisol and corticosterone are secreted from the adrenal cortex, causing an immunosuppressive effect that interferes with the activation of lymphocytes, monocytes, and T lymphocytes. In addition, through the sympathetic-adrenomedullary system, epinephrine (Epi) and norepinephrine (NE) are secreted from adrenal medulla chromaffin cells [51]. Among them, the most commonly used stress-related marker is serum cortisol. One study reported that cognitive behavior therapy (CBT), a psychological treatment technique that can relieve stress in patients with tinnitus, significantly decreased post-treatment serum cortisol levels compared to other treatments. This study argued that using this result, the effect of CBT can be predicted through serum cortisol levels [52]. Another study found that in patients with chronic tinnitus, as the intensity and frequency of tinnitus increased, steroid metabolites, including serum cortisol and pregnenolone, decreased [53]. These results support the theory that, unlike acute stress stimulation, chronic stress stimulation induces an abnormal response of the HPA, resulting in a decrease in the cortisol response because of chronic stimulation compared to that in healthy controls [54].

Chronic stress stimulation disturbs the endocrine system, thereby reducing not only serum cortisol but also adrenocorticotropic hormone (ACTH) and increasing β-endorphin levels [55]. However, in a study of patients with chronic subjective tinnitus, hormone levels, including ACTH, β-endorphin, prolactin, and urinary catecholamine did not change according to the stress score, but ACTH was associated with immunosuppression markers related to stress response [56] In addition, serotonin (5-hydroxytryptamine, 5-HT), synthesized and secreted by the brain in stressful situations, plays an important role in filtering and regulating auditory information, and abnormal serotonin secretion and synthesis are considered to be one of the processes of recognizing tinnitus [57]. When measuring 5-hydroxyindoleacetic acid (5-HIAA), a metabolite of 5-HT, serum cortisol, epinephrine, and norepinephrine, the percentage of people with increased 5-HIAA was higher in the tinnitus group, and 5-HIAA levels were associated with tinnitus duration, NE, and serum cortisol levels. Although the increase in hormone levels was related to the subjective tinnitus evaluation score, it appeared to be an independent risk factor for tinnitus [58].

As such, serum cortisol is considered a representative stress hormone and is used in many studies, but it has a circadian rhythm, which causes an increase in its levels in the morning and decrease in the afternoon, so it is difficult to measure its baseline level through a single blood test. Therefore, saliva specimens have been preferred over blood specimens to measure basal levels of hormones. Although saliva specimens have disadvantages, including the need to collect several samples for accurate measurements of basal hormone levels, these samples are widely used because of their advantages, including non-invasive collection, the laboratory independence of sampling, and lower cost than blood specimens. In particular, because cortisol enters the saliva by passive diffusion, it is not affected by the saliva flow rate. Therefore, to accurately measure the physiologically active cortisol circulating in the body, cortisol has been measured in saliva, which is an inexpensive test, in neuroendocrine studies [59]. As a result of analyzing the baseline cortisol level measured by collecting five saliva samples over three days for a week, a high tinnitus-related distress group showed higher basal cortisol level and intolerance to external auditory stress than that of a low tinnitus-related distress group and control group [60]. The basal cortisol level was chronically elevated in patients with chronic tinnitus, but the increase in cortisol response to stress stimulation was delayed compared to that in the control group [54]. This blunted response reflects an abnormality of the HPA caused by the chronic stress response. Basal steroid levels show negative feedback by binding to the mineralocorticoid receptor, but the negative feedback from the cortisol level due to stress response mainly acts on the glucocorticoid receptor [61]. These glucocorticoid receptors are often found in the inner ear, pituitary gland, brain stem, hippocampus, and amygdala, so it is thought that changes in cortisol caused by stress response act not only on the pituitary gland, but also on the inner ear, inducing sensitivity to external sounds. In a study comparing tinnitus patients with similar age, sex, education level, and health status, basal cortisol levels were similar between the two groups, but the cortisol suppression response by low-dose dexamethasone suppression test through glucocorticoid receptor appeared stronger and longer in patients with tinnitus. It was also found that the hearing discomfort threshold was lowered because sensitivity to external acoustic stimuli was increased [62].

The cortisol awakening response (CAR), which is the cortisol level measured after waking up, reflecting our body’s response to stress, is known to decrease in chronic fatigue syndrome or depression [63] When the CAR was measured in patients with chronic tinnitus, it was observed that patients under chronic stress from tinnitus had lower CAR than those who had adapted to tinnitus or the control group [64]. These studies support the abnormality of the HPA caused by chronic stress and clarify the relationship between tinnitus and stress. In contrast, in a few studies, no change in salivary cortisol level was found in patients with tinnitus, and no significant change in salivary cortisol level was reported after sound therapy [65,66]. In addition, an interesting study using serum melatonin, which was produced in the pineal gland and previously reported to be helpful in the treatment of sleep disorders and tinnitus, showed that the concentration of serum melatonin was significantly low in patients with chronic tinnitus; therefore, melatonin supplementation would be helpful in the treatment of tinnitus in the future [67,68]. In conclusion, the above results show that chronic tinnitus acts as a chronic stress stimulator and affects the secretion of hormones related to the stress response. It also seems to interfere with the normal response to stress by disturbing the normal secretion of hormones in the body. Moreover, since changes in hormone levels are associated with the effect of tinnitus treatment, the treatment effect can be predicted through the endocrine parameters. Therefore, in patients with tinnitus, stress management and tinnitus treatment are required, and endocrine markers may be helpful in establishing a treatment plan.

### 4.5. Immunologic Parameters

Interleukin (IL)-1α, 1β, 2, 6, Tumor necrosis α (TNF α)

IL-10, 12

Heat shock protein 70 (HSP-70)

CD19, CD16NK

Salivary neopterin

As it is widely known that psychiatric stress affects the immune system of the body, studies related to tinnitus and changes in the immune system have been conducted [69]. In particular, chronic tinnitus has attracted more attention because chronic diseases that are difficult to control are the main factors inducing the response of the immune system. The first reported parameters in humans were tumor necrosis factor-α (TNF-α), which is a T helper (Th1) marker, and interleukin (IL)-6 and IL-10, which are Th2 markers that are known to be related to psychiatric stress in humans and have already been studied in animal experiments [70]. The pro-inflammatory cytokines, IL-6 and TNF-α, are increased not only in infectious diseases, but also in major depressive disorders, and TNF-α is increased in chronic central nervous system (CNS) diseases, such as Alzheimer’s disease and multiple sclerosis [71,72]. As a result of conducting a progressive muscle relaxation (PMR) program and regular training at home through psychiatrists for patients with tinnitus, stress perception, anxiety, and discomfort caused by tinnitus, as well as TNF-α, a pro-inflammatory cytokine, were decreased. However, there were no changes in IL-6 and IL-10 levels before and after treatment [73]. In addition, when CBT was administered to patients with tinnitus, the level of IL-2, which is known to be involved in cellular immunity, was increased compared to that in patients who received only sound therapy [52]. Pro-inflammatory cytokines, known to increase in post-traumatic stress disorder (PTSD) and major depressive disorders, are known to regulate synaptic strength and synaptic plasticity in the nervous system, thus so studies involving IL-1β were conducted [74,75]. According to a study that analyzed the association between circulating cytokine and psychometric scores, TNF-α showed a positive correlation with “tinnitus loudness”, “total perceived stress”, “tension” and “depression”, but negative correlation with “joy” scores. Also, IL-1β showed a positive correlation with visual analogue scale (VAS) score awareness” and suggested that cytokine could be used as a diagnostic marker in tinnitus [76]. In a study of tinnitus with presbycusis, TGF-β was low in the elderly, and IL-1α was high in patients with tonal tinnitus, and IL-2, representing cellular immunity, was low in patients with partial or complete residual inhibition. IL-10, an anti-inflammatory cytokine, was lower in patients with tinnitus than in the control group, and decreased as the duration of tinnitus increased. In addition, heat shock protein 70 (HSP-70), which is involved in autoimmune reactions in the inner ear and is known as a prognostic factor for sudden sensorineural hearing loss, decreased as the loudness of tinnitus increased [77].

These results provide evidence for the relationship between tinnitus and the immune response, but the effect of aging cannot be excluded in that presbycusis is included. In a study using lymphocyte subpopulation as an indicator of an immune response, CD19 and CD16NK were found to be related to changes in ACTH levels caused by chronic stress [56]. Another interesting immunologic marker is neopterin, which is produced by monocytes/macrophages and found in body fluid. Application of acute stress has been found to alter serum neopterin concentrations in animals and in patients with chronic tinnitus, and increases in perceived stress scale score were found to reduce salivary neopterin levels [78]. In conclusion, the immune system reaction that occurs in patients with tinnitus is similar to that in other chronic diseases. Among the immunologic markers, pro-inflammatory substances increase and decrease after treatment or residual inhibition, respectively.

### 4.6. Neurologic Parameters

Brain-derived neurotrophic factor (BDNF)

Glial cell line-derived neurotrophic factor (GDNF)

Blood serotonin

5-HTTLPR polymorphism in SLC6A4

Glutamate metabotropic receptor 7 (GRM7)

Salivary α-amylase

Serum magnesium

As mentioned in the introduction, changes in the CNS in the development and maintenance of tinnitus are considered the main mechanisms of tinnitus, and there are several neurological theories to explain this. These include (1) neurophysiological theory, which is the basis of tinnitus retraining therapy (TRT), (2) spontaneous firing rate theory, which refers to the hyperexcitability of the central auditory region because of the decrease in the inhibitory cortical process in the cerebral cortex as a result of hearing loss, and (3) neural synchrony theory, which states that tinnitus occurs because of simultaneous neural ignition in several local neurons as the tonotopy changes from the damaged frequency region in the auditory center to the normal frequency region as a result of hearing loss. Taken together, in almost all forms of cochlear injury, the nerve response transmitted to the auditory center decreases initially, but. Still, as goes by, nerve ignition increases in the auditory nerve pathway, and synchronization with the overactivity of the central auditory nervous system is achieved. An increase in excitatory signals along the central auditory pathway because of a decrease in inhibitory nerve input signals, deafferentiation of the auditory nervous system, and neural plasticity, such as the formation of new synapses as a result of nerve ignition or changes in synaptic structure can induce tinnitus [12,13,15,79]. Therefore, studies have been conducted to use the factors related to neuroplasticity in the central auditory system as an objective biomarker for the diagnosis, evaluation, and treatment of tinnitus. Brain-derived neurotrophic factor (BDNF) is a type of nerve growth factor (NGF) that belongs to the neurotrophic protein family and helps in nerve growth, differentiation, and survival in the central and peripheral nervous systems [80]. Therefore, BDNF plays a major role in synaptic plasticity and neurogenesis. In animal studies, it has been found that BDNF expression in the inner ear increases when tinnitus is induced [81]. In addition, it is secreted in the brain through regular exercise, and secretion decreases in stress-related diseases, such as major depressive disorders [82]. BDNF is detected in both brain and peripheral blood, but since BDNF passes through the blood–brain barrier (BBB), studies are mainly conducted using serum BDNF rather than brain BDNF [83]. Plasma BDNF levels are related to tinnitus severity. When patients with tinnitus were classified as mild or severe according to the tinnitus handicap inventory (THI) score, those with mild tinnitus had higher plasma BNDF levels than those with severe tinnitus and the control group, and patients with lower hospital anxiety and depression scale (HADS) scores had low THI scores and high plasma BDNF levels [10]. In another study, plasma BDNF levels in patients with tinnitus were higher than those in the control group, and decreased after TRT in patients with severe tinnitus [84]. Plasma BDNF levels showed significant results in most studies, but were not related to the psychometric score [76]. Glial cell line-derived neurotrophic factor (GDNF) is another neurotrophic factor involved in the development of the central auditory system and inner ear, as well as nerve growth, differentiation, and survival with BDNF. Therefore, on the basis of the importance of these two factors, a study on the coding genes of both factors was conducted. The BDNF gene is approximately 70-kB long and is located in the short arm of chromosome 11 (11p14.1), and the GDNF gene is located in 5p13.2 [85].

The most well-known genetic variation discovered through the study of gene polymorphism is BDNF single-nucleotide polymorphism (SNP) rs6265 (known as BDNF Val66Met), where guanine (G) is changed to adenine (A) and the 66th amino acid is changed from valine to methionine. Until now, BDNF Val66Met is known to be associated with psychiatric diseases, such as schizophrenia, major depressive disorders, and CNS diseases, including Parkinson’s disease and Alzheimer’s disease. In otology, BDNF gene SNPs, rs6265, rs2030324, and rs1491850 were found to be associated with low serum BDNF levels and changes in auditory evoked potential [10,86,87,88,89]. In one study on GDNF and BDNF gene SNPs, there was no allelic association between two BDNF SNPs (rs6265, rs2049046) and three GDNF SNPs (rs1110149, rs884344, rs3812047) in patients with chronic subjective tinnitus. However, the combination of BDNF and GDNF gene polymorphisms was more associated with the severity of tinnitus in women than in men [90]. Another study reported that the plasma BDNF level was low in patients with tinnitus, but the BDNF gene polymorphism was not different from that of the control group [91]. Similarly, when comparing the GDNF gene polymorphisms (rs884344, rs3812047, and rs1110149) between patients with chronic tinnitus and the control group, the heterozygosity of rs1110149 was lower in patients with tinnitus, but there was no difference in the allele frequency of the three polymorphisms [92]. Therefore, focusing on the difference in expression level rather than the difference in gene polymorphism, the methylation patterns of peripheral blood specimens were used instead of the methylation patterns of brain tissue [93]. The CpG sites are deoxyribonucleic acid (DNA) regions in which cytosine and guanine nucleotides exist in succession, and are sites that can regulate gene expression through methylation of cytosine. In fact, a comparison of the methylation of 12 CpG sites in the BDNF and GDNF promoter regions in patients with chronic tinnitus and the control group, there was a difference in the methylation ratio of BDNF CpG6 and GDNF CpG3-5-6 between the two groups [94]. Serotonin is an important neurotransmitter that is mainly present at the nerve endings in the central and peripheral nervous systems and plays an important role in the activation of the limbic and reticular brain structures that regulate the contraction and dilatation of blood vessels. Therefore, since serotonin is involved in the regulation of blood flow through the CNS, a study on the amount of serotonin in the blood was conducted. In about 67% of patients with tinnitus, the blood serotonin concentration was higher than the reference value, but there was a limitation in that the number of subjects was small [95]. Furthermore, since nerve fibers containing serotonin are mainly located in auditory pathways, including auditory nuclei and inferior colliculus, it was considered that serotonin modification would contribute to auditory signal transmission and the occurrence of tinnitus [96]. Therefore, a study was conducted on the serotonin transporter gene (SLC6A4) with various numbers of tandem repeats (VNTR) naturally present in the promoter region (5-HTTLPR) and second intron.

In a study that analyzed SLC6A4 polymorphism in patients with chronic subjective tinnitus and a control group, there was no difference between the two groups, but a correlation between 5-HTTLPR polymorphism and VAS score was found in patients with tinnitus [97]. In addition, no association was found between BDNF Val66Met and 5-HTTLPR, which are known to be associated with mood disorders in patients with tinnitus, but depressive symptoms were associated with the 5-HTTLPR *S*/*S* genotype in tinnitus, similar to that in other diseases [98].

There are biomarker studies related to serotonin as well as glutamate, a major neurotransmitter in the central and peripheral auditory nervous system. Glutamate acts as an excitatory neurotransmitter in the auditory signaling system, and its main receptors include N-methyl-D-aspartate (NMDA) and alfa-amine propionic acid (AMPA). As studies on these glutamate receptors and tinnitus have been conducted, it has been found in animal models that injecting NMDA antagonists into the cochlear fluid can prevent salicylate-induced tinnitus [99]. Glutamate metabotropic receptor 7 (GRM7) is a gene encoding metabotropic glutamate receptor subtype 7 (mGluR7) and is involved in glutamate synaptic transmission in the CNS by binding to glutamate. In particular, mGluR7 was found in the inner and outer hair cells, and in the spiral ganglion nerve. Therefore, because the association between the SNP of GRM7 and aging of the auditory nervous system was revealed, studies using GRM7 were also conducted [100]. According to a study that targeted patients with presbycusis, whose discrimination threshold was higher than 40 dB sound pressure level (SPL) at 1kHz or higher, when the genotype of the GRM7 rs11928865 SNP was analyzed, the frequency of the T/T genotype was higher than that of the A/T genotype, and the T/T genotype had a 33% lower risk of tinnitus. In addition, the severity of tinnitus and the GRM7 genotype were associated, and the percentage of patients with the A/T genotype was high in the severe tinnitus group. [101]. Although α-amylase is normally found in blood specimens, salivary α-amylase (sAA) is mainly used as a surrogate marker for sympathetic nervous system activity in studies of tinnitus because secretion from the salivary gland is regulated by direct sympathetic innervation. When stress stimulation was administered to male patients with tinnitus and the control group, the sAA levels were lower in patients with tinnitus than in the control group, and the sAA levels were lower as the perceived stress scale (PSS) was higher. This result indicates that the sympathetic activity, as well as the abnormality of HPA, are impaired by chronic stress stimulation caused by tinnitus [65]. A study on serum magnesium, which is known to help treat sudden sensorineural hearing loss and acts as a regulator of the calcium channel in the neurotransmission process, showed lower serum magnesium levels in patients with tinnitus than tinnitus than in the control group [102,103].

In conclusion, the above results suggest that chronic tinnitus stimulation causes damage to the nervous system as well as the endocrine system, and serves as evidence for the development of tinnitus through neuroplasticity. In addition, since it suggests that not only the difference in protein levels but also the genetic variation and expression difference are involved in the pathophysiology of tinnitus, it is believed that the genetic evaluation of many biomarkers will be necessary in future studies.

### 4.7. Oxidative Parameters

Serum total oxidant status (TOS), total antioxidant status (TAS), oxidative stress index (OSI), Paraoxonase (PON)

Lipid hydroperoxide (LOOH), Thiol/Disulphide homeostasis (TDH)

Nitric oxide, glutathione peroxide (cGPx), glutathione S-transferase (GST), superoxide dismutase (SOD)

Plasma Malondialdehyde (MDA), 4-hydroxynonenal (4-HNE)

N-acetyltransferase (NAT2)

Vitamin B12

Serum Zinc

Plasma apelin

Oxygen molecules are converted into highly reactive free radical intermediates during metabolism in the body, called reactive oxygen species (ROS). Because ROS damage cellular components, such as lipids, proteins, and DNA, they can affect various tissues in the body. Although our body has a defense mechanism against these free radicals, the damage caused by free radicals is called ‘oxidative stress’ [104]. Therefore, through studies on various diseases, including otologic diseases, such as Meniere’s disease, it has been found that oxidative stress is involved in the pathophysiology of several diseases [105]. Oxidative stress is believed to be involved in the pathophysiology of tinnitus, as it is known to cause hair cell death, cochlear degeneration, and changes in the stria vascularis of the cochlear duct, as well as neural activity [106,107]. According to a study using total oxidant status (TOS), total antioxidant status (TAS), oxidative stress index (OSI), and paraoxonase (PON), which are oxidative stress markers traditionally used as indicators of oxidant/antioxidant balance, TAS and PON of patients with tinnitus were lower than those of the control group, but TOS and OSI were higher [108]. Although the number of subjects was small, the study of idiopathic tinnitus with normal hearing also showed higher TOS and OSI in patients with tinnitus than that in the control group, but there was no difference in TAS [109].

Another antioxidant system, thiol-disulfide homeostasis (TDH), is composed of a cycle of thiols (-sulfhydryl groups) and disulfide compounds, which form when thiols meet free radicals [110]. In a previous study comparing lipid hydroperoxide (LOOH) and TDH in young patients with tinnitus with normal hearing and the control group, TOS and OSI were high in patients with tinnitus, and TAS was low. Native thiol and native thiol/total thiol ratios were low in patients with tinnitus, and disulphide, disulphide/native thiol ratio, and disulfide/total thiol ratio were high. In addition, LOOH, an oxidative stress marker, was high in patients with tinnitus [111]. Various substances are related to oxidants and antioxidants, including malondialdehyde (MDA) as a representative substance produced by the oxidation of unsaturated lipids like LOOH, superoxide dismutase (SOD) as endogenous antioxidant enzymes, catalase, glutathione peroxidase (cGPx), glutathione S-transferase (GST), glutathione reductase (GR), and ceruloplasmin. A comparison of the above enzymes and proteins in patients with idiopathic tinnitus and the control group showed that cGPx and SOD activity were lower in patients with tinnitus, but GST activity was higher. The concentrations of MDA and nitric oxide, which are oxidative stress markers, were higher, and there was no difference in ceruloplasmine oxidase activity [112]. This means that the antioxidant system does not work effectively in patients with tinnitus. In addition, N-acetyltransferase (NAT) activity according to the genotype of the NAT gene, which acts as an antioxidant through O-acetylation, is related to the severity of tinnitus [101]. However, there is no unified result regarding the effects of antioxidative substances on tinnitus. When several antioxidative substances (multivitamin and lipoic acid), which are effective against tinnitus in animal studies, were applied to patients with chronic tinnitus, the intensity and loudness of tinnitus decreased, but objective markers, such as total antioxidant capacity (TAC), SOD, and oxidized low-density lipoprotein LDL (oxLDL) were not changed [113].

However, in studies using phospholipids and vitamins (glycerophosphorylcho-line, glycerophosphorylethanolamine, β-carotene, vitamin C, and E), levels of ROS, such as MDA and 4-hydroxynonenal (4-HNE) decreased, and tinnitus-related discomfort decreased [114]. In fact, studies measuring antioxidative substance levels in patients with tinnitus also showed variable results. Vitamin B12 levels were higher in the control group than in the patients with tinnitus, but ascorbic acid levels did not differ between the two groups. Serum zinc levels are associated with tinnitus severity [68,115]. Recently, research on apelin, which is derived from the vascular endothelium and adipocytes, showed that it prevents damage to blood vessels caused by oxidative stress in cardiovascular disease. In fact, since the apelin level of patients with tinnitus was low and decreased as the THI score increased, it is expected to be helpful in the treatment of tinnitus [28]. In conclusion, although the above results do not show the damaged site where oxidative stress occurs, oxidative stress is involved in the pathophysiology of tinnitus, similar to that observed in other diseases. Therefore, more research on the effectiveness of antioxidant therapy is needed, and it is expected to be helpful in the treatment of tinnitus.

### 4.8. Other Markers

Serum prolidase

Saliva/Urine cotinine

Alkaline phosphatase (ALP), Erythrocyte

Prolidase, which belongs to the family of hydrolases and is involved in collagen degradation and synthesis, has been demonstrated as a causative factor in various diseases, such as nasal polyps and asthma [116,117]. In a study of 25 patients with chronic tinnitus with normal hearing, low serum prolidase enzyme activity was noted in patients with tinnitus [109]. This is considered to be evidence of oxidative stress in tinnitus, considering the relationship between proline and oxidative stress observed in animal studies. A study on smoking and tinnitus was conducted based on data from the National Health and Nutrition Survey in Korea. Similar to blood specimens, urine specimens are collected for routine examination. In contrast to blood specimens, however, urine specimens are collected non-invasively, providing a distinct advantage. Cotinine has a longer half-life than nicotine, making urinary cotinine a measurable biomarker for tobacco smoking, especially in patients with chronic diseases, such as chronic kidney disease (CKD). Although there was no exact criterion for the duration of tinnitus, the study reported that urine cotinine acts as an independent risk factor in patients with tinnitus and that smoking amount is associated with tinnitus-related annoyance [118]. Secondhand smoke in children is also associated with hearing loss. A study in pediatric patients with tinnitus showed that the patient’s saliva cotinine, parents’ saliva cotinine, alkaline phosphate (ALP), and erythrocytes increased the risk of tinnitus. In addition, the higher the parental saliva cotinine level, the higher was the saliva cotinine level in pediatric patients with tinnitus [119].

## 5. Conclusions

**Figure 2:**
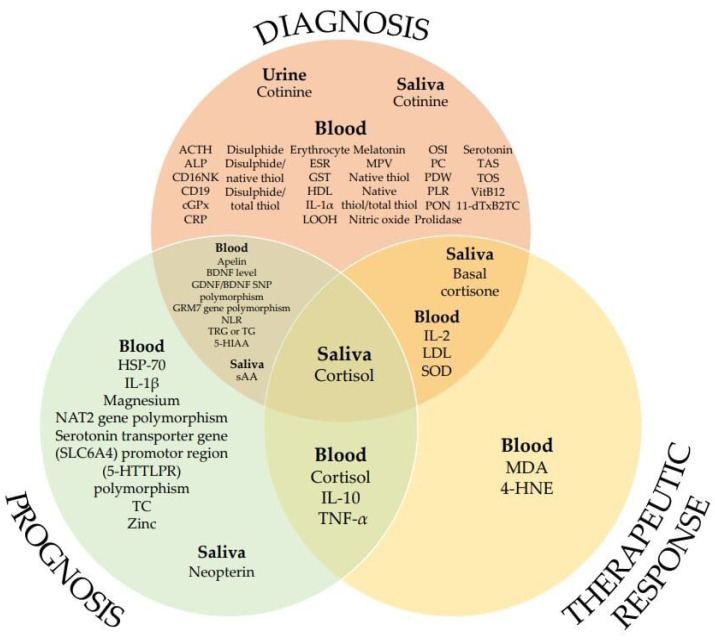
List of biomarkers according to potential applications (in no particular order).

Figure 2 shows the biomarkers used as indicators for diagnosis, prognosis, and therapeutic response in patients with chronic subjective tinnitus.

These are classified into eight categories according to their characteristics, including metabolic, hemostatic, inflammatory, endocrine, immunologic, neurologic, oxidative, and other parameters (Table 1).

Of the total 58 biomarkers, the most frequently studied markers were MPV in hemostatic parameters and NLR in inflammatory parameters, and saliva cortisol in endocrine parameters had the most diverse potential applications. According to the hypothesis of tinnitus pathophysiology, diverse factors in various fields, such as the nervous system and stress-related neuropsychiatry are involved in the occurrence of tinnitus. Studies to identify biomarkers that can objectively measure and evaluate tinnitus have been conducted in various fields (Table 2). In the future, an individualized and diversified approach is needed for the research on tinnitus, including the clinical field involving the diagnosis and treatment of tinnitus, rather than a standardized method.

## Figures and Tables

**Table 1:** Biomarkers suggesting prognosis and severity in chronic subjective tinnitus.

	Biomarkers
Endocrine parameters	• Increase Saliva basal cortisol/cortisone (with blunted response to stress), 5-HIAA • Decrease Serum cortisol, Plasma melatonin level • ACTH (related with CD16NK, CD19)
Hemostatic parameters	• Increase MPV (related with HFHL), PDW, PC, 11-dTxB2
Immunologic parameters	• Increase IL-1α (related with tonal tinnitus), IL-1β, IL-2, TNF-α • Decrease IL-10, HSP-70, Saliva neopterin • CD16NK, CD19 (related with ACTH)
Inflammatory parameters	• Increase NLR (related with HFHL-TN), PLR, CRP, ESR
Metabolic parameters	• Increase TC, TG or TRG, LDL • Decrease HDL
Neurologic parameters	• Increase Plasma BDNF level, Blood serotonin level • Decrease Saliva α-amylase, Serum magnesium • BDNF/GDNF SNP polymorphism, Serotonin transporter gene (SLC6A4) promoter region (5-HTTLPR) polymorphism, GRM7 gene polymorphism (relative with severity)
Oxidative parameters	• Increase TOS, OSI, LOOH, Nitric oxide, GST, MDA, 4-HNE, Disulphide, Disulphide/native thiol ratio, Disulphide/total thiol ratio • Decrease TAS, PON, cGPx, SOD, Native thiol, Native thiol/total thiol ratio, Vit B12, Plasma apelin level, Serum zinc • N-acetyltransferase 2 (NAT2) gene polymorphism (relative with severity)
Others	• Increase Saliva/Urine cotinine, ALP, Erythrocyte • Decrease Serum prolidase

Abbreviations: TC: Total cholesterol; TRG: Triglyceride; LDL: Low-density lipoprotein; HDL: High-density lipoprotein; N/A: Not applicable; NLR: Absolute count of neutrophils/lymphocyte; PLR: Abscolute count of platelet/lymphocyte; MPV: Mean platelet volume; BNDF: Brain-derived neurotrophic factor; SNP: Single nucleotide polymorphism; ALP: Alkaline phosphatase; 11-dTxB2: 11-dehydro-thromboxane B2; TAS: Total antioxidant status; TOS: Total oxidants status; OSI: Oxidative stress index; TG: Triglyceride; IL-2: Interleukin-2; SOD: Superoxide dismutase; IL-1α: Interleukin-1α; IL-10: Interleukin-10; TNF-α: Tumor necrosis factor-1α; HSP70: Heat shock protein-70; GDNF: Glial-derived neurotrophic factor; MDA: Malondialdehyde; cGPx: Glutathione peroxidase; GST: Glutathione S-transferase; LOOH: Lipid hydroperoxide; PC: Platelet count; PDW: Platelet distribution width; GRM7: Glutamate metabotropic receptor 7; NAT2: N-acetyltransferase 2; PON: Paraoxonase-1; ESR: Erythrocyte sedimentation rate; CRP: C-reactive protein; IL-1β: Interleukin-1β; IL-6: Interleukin-6; 5-HIAA: 5-hydroxyindoleacetic acid; 5-HTTLPR; 44-bp insertion-deletion in the promoter region; ACTH: Adrenocorticotropic hormone; 4-HNE: 4-hydroxynonenal.

**Table 2:** Summary of biomarkers for chronic subjective tinnitus.

Author (Year)	Study Design	Subjectives	Ages (years)/Hearing State	Specimen/Tissue Type	Biomarkers	Potential Application	Results/Conclusions
Deniz Avci (2021) [29]	Retrospective, case-control study	91 patients, 65 controls (age-,sex-matched)	18–70 years/ Normal hearing	Blood	Metabolic parameters (TC, TRG, LDL, HDL)	Diagnosis	Mean TC, TRG, and LDL levels were higher in the tinnitus group.
Demir M (2021) [35]	Retrospective, case-control study	159 patients, 57 controls (age-,sex-matched)	21–67 years/ N/A	Blood	Hemostatic parameters (MPV) Inflammatory parameters (NLR, PLR)	Diagnosis	AFHL-TN group had higher NLR than that of CNT group; HFHL-TN group had higher PLR than that of AFHL-TN, NH-TN, and CNT groups; HFHL-TN had higher MPV than that of AFHL-TN, NH-TN, and CNT groups.
Jeong et al. (2021) [98]	Case-control study	86 patients, 252 controls	22–83 years/ Normal hearing (better than 30 dB in 250 to 6000 Hz)	Blood	Neurologic parameters (BDNF gene SNP rs6265 [Val66Met], Serotonin transporter gene-linked polymorphic region 5-HTTLPR)	Prognosis	Depressive symptom in tinnitus is associated with the 5-HTTLPR S/S genotype and the severity of tinnitus.
Ali Seyed Resuli (2020) [119]	Retrospective, case-control study	415 children patients, 200 controls (age-matched)	7–15 years/ Normal hearing	Blood/Saliva	Others (Saliva cotinine, ALP, Erythrocyte)	Diagnosis	Saliva cotinine level of children and their parents, serum erythrocyte and serum ALP level are associated with tinnitus.
Yildiz et al. (2020) [34]	Retrospective, case-control study	287 patients, 275 controls	18–59 years/ Normal hearing (Pure tone average lower than 20 dB)	Blood	Hemostatic parameters (MPV) Inflammatory parameters (NLR)	Diagnosis	The percentage of subjects with high MPV and mean NLR level was higher in the tinnitus group. NLR level of 2.17 and above is associated with 1.991 times higher risk of tinnitus.
Chrbolka et al. (2020) [39]	Prospective cohort study	40 patients, 40 controls (age-,sex- matched)	Mean: 50.86 years/ Hearing loss (Pure tone average lower than 40 dB)	Blood	Hemostatic parameters (11-dTxB2)	Diagnosis	Tinnitus patients have higher levels of 11-dTxB2 and increased platelet activity.
Ekinci A et al. (2020) [109]	Case-control study	25 patients, 25 controls	18–65 years/ Normal hearing	Blood	Oxidative parameters(TAS, TOS, OSI) Others (Prolidase)	Diagnosis	TOS, OSI and prolidase activity level were higher in tinnitus patients.
James George Jackson (2019) [64]	Case-control study	20 patients, 10 controls	Mean: 52.2 years/ N/A	Saliva	Endocrine parameters (Cortisol)	Prognosis	Changes in cortisol levels—the AUCi—were less robust in severe tinnitus and AUCi correlated negatively with tinnitus distress later the same day.
Aydin N et al. (2019) [66]	Prospective study	21 patients	22–69 years/ N/A	Saliva	Endocrine parameters (Cortisol, Cortisone)	Therapeutic response (Sound therapy)	No changes in salivary cortisol, cortisone following sound therapy.
Ensari N et al. (2019) [28]	Case-control study	40 patients, 40 controls	More than 18 years/Normal hearing	Blood	Oxidative parameters (Plasma apelin) Metabolic parameters (HDL, TG)	Diagnosis/Prognosis	Mean plasma apelin level, HDL were lower, but TG level was higher in patients with tinnitus. Negative correlations between apelin and THI were found.
Gunes A et al. (2019) [48]	Prospective study	52 patients	35–50 years/ N/A	Blood	Hemostatic parameters (MPV) Inflammatory parameters (NLR)	Prognosis	Correlation was observed between high-frequency PTA, high-frequency DPOAE, and NLR.
Li J et al. (2019) [52]	Case-control study	100 patients	18–83 years/ N/A	Blood	Endocrine parameters (Serum cortisol) Immunologic parameters (IL-2)	Therapeutic response (CBT)	Serum cortisol level was decreased and IL-2 level was increased after CBT
Petridou A I et al. (2019) [113]	Case-control study	70 patients	25–75 years/ Normal hearing or up to moderate hearing loss	Blood	Oxidative parameters (TAC, SOD, oxLDL)	Therapeutic response (Antioxidant)	No changes in TAC, SOD, and oxLDL were found after antioxidant supplementation (Multivitamin, alpha-lipoic acid).
Haider H F et al. (2019) [77]	Case-control study	92 patients, 22 controls	55–75 years/ N/A	Blood	Immunologic parameters (IL-1α, IL-2, IL-10, TNF-α HSP70)	Diagnosis/Prognosis	IL-10 levels were lower in patients with tinnitus, but IL-1α was higher in tonal tinnitus. IL-2 was lower in patients with residual inhibition. Negative correlation between tinnitus duration and IL-10, and between HSP70 and tinnitus loudness.
Orenay-Boyacioglu S et al. (2019) [94]	Case-control study	60 patients, 50 controls (Age-matched)	18–55 years/ Normal hearing	Blood	Neurologic parameters (BDNF/GDNF CpG promoter methylation)	Diagnosis	BDNF CpG6 and GDNF CpG3-5-6 methylation ratios were different between controls and tinnitus group.
Yuksel F et al. (2018) [27]	Retrospective study	215 patients	18–75 years/ N/A	Blood	Metabolic parameters (TC, LDL, TG)	Prognosis	Increased IMT in tinnitus was associated with the severity of tinnitus (THI, VAS), high levels of TC, LDL, TG.
Lee et al. (2018) [118]	Cross-sectional study	486 adolescents patients, 2296 controls	12–18 years/ Normal hearing (lower than 40 dB at 500, 1000, 2000 and 3000 Hz)	Urine	Others (Cotinine)	Diagnosis	Urine cotinine level was the only parameter associated with tinnitus and the amount of smoking with tinnitus-related annoyance. Urine cotinine may be a biomarker for treatment response in adolescents with tinnitus
Pawlak-Osinska K et al. (2018) [112]	Case-control study	51 patients, 19 controls	20–62 years/ N/A	Blood	Oxidative parameters (Cp, GSH, Nitrate/nitrite, MDA, cGPx, GST, SOD-1)	Diagnosis	As antioxidants, cGPx, SOD were lower in tinnitus group, but GST activity was higher. GSH, MDA, and nitric oxide were higher in tinnitus group, but Cp showed no significant results. Antioxidants barrier system showed inefficiency in tinnitus group.
Celik M et al. (2018) [111]	Prospective case-control study	35 patients, 35 controls (age-sex-BMI-matched)	27–56 years/ Normal hearing	Blood	Oxidative parameters (LOOH, TAS, TOS, OSI, Thiol/Disulphide)	Diagnosis	In patient group, TOS and OSI levels were higher and TAS levels were lower. Disulphide and disulphide/native thiol and disulphide/total thiol ratio were higher in patient group, but native thiol levels and native thiol/total thiol ratio were lower. Also, LOOH ratio was higher
Düzenli U et al. (2018) [49]	Retrospective case-control study	58 patients, 58 controls	Mean: 38.8 years/ Normal hearing	Blood	Hemostatic parameters (MPV, PC, PDW) Inflammatory parameters (NLR)	Diagnosis	PDW was higher, but NLR was lower in tinnitus group. Also, MPV values were similar between tinnitus group and controls. There were no correlation between hematologic and audiometric values.
Ulusoy B et al. (2018) [37]	Prospective case-control study	64 patients, 64 controls (age-matched)	18–65 years/ N/A	Blood	Hemostatic parameters (MPV, PDW) Inflammatory parameters (WBC, NLR, PLR)	Diagnosis	WBC, MPV, and PDW levels were higher in tinnitus, but no significant results were observed for NLR and PLR. Prothrombotic condition plays a role in the pathophysiology.
Chrbolka P et al. (2017) [53]	Cross-sectional study	28 patients	Mean (male 52.5, female 55.2 years)/ Normal hearing (pure tone average lower than 40dB)	Blood	Endocrine parameters (Neuroactive, neuroprotective, immunomodulatory steroids)	Prognosis	Negative correlation between tinnitus indices and intensity of steroidogenesis. Also, circulating steroids negatively correlated with degree of tinnitus because of HPA abnormality.
Bayraktar C et al. (2017) [46]	Prospective case-control study	40 patients, 40 controls (age-sex-matched)	Mean 41 years/ Normal hearing	Blood	Inflammatory parameters (NLR)	Prognosis	Positive correlation between the severity of tinnitus and CCA stiffness index, YEM measurements, left CIMT, and NLR.
Coskunoglu A et al. (2017) [91]	Case-control study	65 patients, 42 controls (age-matched)	18–55 years/ N/A	Blood	Neurologic parameters (Plasma BDNF, BDNF gene SNP rs6265 [Val66Met], rs2030324, rs1491850)	Diagnosis	Serum BNDF level was lower in tinnitus group, but no correlation BDNF gene polymorphism with tinnitus.
Haider H F et al. (2017) [101]	Cross-sectional study	50 patients, 28 controls	55–75 years/ Sensory presbycusis (bilateral SNHL in downslope audiometric pattern, above 1kHz with poor speech discrimination.	Blood	Neurologic parameters (GRM7 SNP rs11928865) Oxidative parameters (NAT2 SNP)	Diagnosis/Prognosis	Patients with T/T genotype at GRM7 had 33% lower risk for tinnitus and A/T genotype had a tendency for increased severity of tinnitus. Also, the odds of developing severe tinnitus were higher in the slow acetylator phenotype of NAT2. Therefore, Genotype A/T at GRM7 and slow acetylator NAT2 are prone to developing a more severe tinnitus.
Koc S et al. (2016) [108]	Prospective case-control study	54 patients, 60 controls	18–65 years/ Normal hearing	Blood	Oxidative parameters (PON, TOS, TAS, OSI)	Diagnosis	TAS, PON were lower, but TOS, OSI were higher in tinnitus. Tinnitus group was exposed to oxidative stress.
Sarikaya Y et al. (2016) [38]	Prospective case-control study	101 patients, 54 controls (age-sex-matched)	Mean 40.87 years/ Normal hearing	Blood	Hemostatic parameters (MPV)	Diagnosis	MPV level was increased in tinnitus, but no significant association between MPV level and duration or characteristics of tinnitus.
Uluyol S et al. (2016) [103]	Case-control study	76 patients (severe and catastrophic tinnitus), 86 controls	43–65 years/ Normal hearing	Blood	Neurologic parameters (Serum magnesium)	Prognosis	Serum magnesium concentration was lower in severe tinnitus group.
Xiong H et al. (2016) [84]	Restrospective case-control study	82 patients, 32 controls	Mean 42.7 years/ N/A	Blood	Neurologic parameters (Plasma BDNF)	Diagnosis/ Therapeutic response (TRT)	Plasma BDNF levels were higher in tinnitus and decreased after effective TRT, but there was no correlation with tinnitus loudness and severity.
Yuksel F et al. (2016) [36]	Case-control study	100 patients, 100 controls (age-sex-matched)	Mean 50.95 years/ N/A	Blood	Hemostatic parameters (MPV, PDW, PC) Inflammatory parameters (CRP, ESR)	Diagnosis	MPV was lower, but PC and PDW were higher in tinnitus. ESR and CRP did not show any significant results.
Alsalman O A et al. (2016) [65]	Case-control study	10 patients (male), 10 controls	18–35 years/ Normal hearing	Saliva	Endocrine parameters (Cortisol) Immunologic parameters (Neopterin) Neurologic parameters (sAA)	Diagnosis/Prognosis	sAA levels were lower in tinnitus group, suggesting impaired sympathetic activity, and stress measurements were negatively correlated with measures of sAA and neopterin.
Orenay-Boyacioglu, S et al. (2016) [92]	Case-control study	52 patients, 42 controls (age-matched)	18–55 years/ N/A	Blood	Neurologic parameters (GDNF gene SNP rs884344, rs3812047, rs1110149)	Diagnosis	Heterozygosity was lower for GDNF rs1110149 in tinnitus, but no correlation was observed between tinnitus and GDNF gene polymorphism.
Bayram A et al. (2015) [40]	Retrospective case-control study	51 patients, 42 controls (age-sex-matched)	Mean 43.47 years/ Normal hearing	Blood	Hemostatic parameters (MPV) Inflammatory parameters (NLR, PLR)	Diagnosis	No significant differences in NLR, PLR, and MPV between tinnitus group and control group.
Kemal O et al. (2015) [33]	Retrospective case-control study	86 patients, 84 controls	Under 50 years/ Normal hearing	Blood	Hemostatic parameters (MPV)	Diagnosis	MPV values were significantly higher in tinnitus group.
Berkiten G et al. (2015) [115]	Cross-sectional study	100 patients	17–78 years/ Non-presbycusis (above 65 years and symmetrical increase in hearing threshold)	Blood	Oxidative parameters (Serum Zinc)	Prognosis	Zinc levels decrease as age increases and were significantly lower in group III (patients between 61–78 years). Also, severity and loudness of tinnitus and audiologic threshold of air conduction were greater in zinc-deficient group.
Ozbay I et al. (2015) [45]	Prospective case-control study	107 patients, 107 controls (age-sex-matched)	Mean 38.7 years/ Normal hearing up to mild hearing loss	Blood	Inflammatory parameters (NLR)	Diagnosis	Despite other hematologic parameters showed no significant results, NLR was higher among the patients.
Szczepek A J et al. (2014) [76]	Prospective study	30 patients	18–67 years/ N/A	Blood	Neurologic parameters (Plasma BDNF level) Immunologic parameters (IL-1β, IL-6, TNF-α)	Prognosis	A positive correlation was observed between TNF-α, tinnitus loudness, total perceived stress, tension, and depression, but a negative correlation with a psychometric score ‘joy’. IL-1β concentration was correlated with awareness grade of tinnitus. No correlation was found between plasma BDNF level and psychometric scores.
Kim et al. (2013) [58]	Case-control study	344 patients, 89 controls	Mean 53.8 years/ N/A	Blood	Endocrine parameters (Cortisol) Neurologic parameters (NE, Epi, 5-HIAA)	Prognosis	The percentage of patients with high 5-HIAA level was higher in tinnitus group, and 5-HIAA levels showed correlation with duration of tinnitus, NE, and cortisol. Elevation of stress-related hormones as well as BDI and BEPSI were the related factors with tinnitus.
Sand et al. (2012) [90]	Cross-sectional study	240 patients	Mean 50.3 years/ N/A	Blood	Neurologic parameters (BDNF gene SNP rs6265 [Val66Met], rs2049046, GDNF gene SNP rs884344, rs1110149, rs3812047)	Prognosis	No significant allelic association but, GDNF and BDNF genotypes jointly predicted tinnitus severity in women.
Simoen V L et al. (2012) [62]	Prospective case-control study	21 patients, 21 controls	Mean 65.7 years/ N/A	Saliva	Endocrine parameters (Cortisol)	Diagnosis/Prognosis	Basal cortisol level was similar, but stronger and longer-lasting cortisol suppression was observed after DEX administration in patients with tinnitus. And discomfort threshold was lower after cortisol suppression.
Goto et al. (2012) [10]	Case-control study	43 patients, 30 controls	Mean 57.1 years/ N/A	Blood	Neurologic parameters (Plasma BDNF level)	Prognosis	Plasma BDNF level was higher in mildly handicapped tinnitus group. Patients with HADS scores ≤14 had lower THI and higher BDNF levels.
Lasisi A O et al. (2012 [68]	Prospective case-control study	81 patients, 58 controls	60–98 years/ N/A	Blood	Endocrine parameters (Plasma melatonin) Oxidative parameters (Ascorbic acid, Vit B12)	Diagnosis	Low plasma melatonin and Vitamin B12 have correlation with tinnitus in elderly, but no significant results were observed with Vitamin C.
Deniz M et al. (2009) [97]	Case-control study	54 patients, 174 controls	20–51 years/ Normal hearing (Pure tone average lower than 30dB at 250Hz to 6kHz)	Blood	Neurologic parameters (Serotonin transporter gene polymorphism [5-HTTLPR, VNTR])	Prognosis	‘*ll*’ genotype of the SLC6A4 polymorphic promoter region was associated with the VAS score, but not with psycho-acoustic parameters in tinnitus.
Hebert S et al. (2007) [54]	Prospective case-control study	18 patients, 18 controls (age-matched)	Mean 68.8 years/ N/A	Saliva	Endocrine parameters (Cortisol)	Diagnosis/Prognosis	There was a blunted cortisol response to psychological stress in tinnitus group compared to that of controls.
Savastano M et al. (2007) [56]	Cross-sectional study	85 patients	18–65 years/ Normal hearing	Blood	Endocrine parameters (Cortisol, ACTH, β-endorphin, prolactin, urinary catecholamine) Immunologic parameters (CD19, CD16NK)	Diagnosis	There was no significant correlation between endocrine parameters and psychological test scores, but lymphocyte CD19 and CD16NK showed correlation with ACTH.
Savastano M et al. (2007) [114]	Cross-sectional study	31 patients	37–72 years/ N/A	Blood	Oxidative parameters (MDA, 4-HNE)	Therapeutic response (Antioxidant)	ROS, including MDA and 4-HNE were reduced following antioxidant treatment, with improvement of VAS and decrease in loudness.
Hebert S et al. (2004) [60]	Case-control study	18 patients, 18 controls	Mean 65.5 years/ N/A	Saliva	Endocrine parameters (Cortisol)	Prognosis	High tinnitus-distress group has elevated chronic cortisol levels, and greater intolerance to external sound.
Weber C et al. (2002) [73]	Case-control study	26 patients, 13 non-tinnitus, 18 controls	20–65 years/ N/A	Blood	Immunologic parameters (IL-6, IL-10, TNF-α)	Therapeutic response (Relaxation program)	TNF-α was reduced after the relaxation program with stress perception, anxiety, anger, and disturbance, but no significant results were observed for IL-6 and IL-10.
Sachanska T. (1999) [95]	Case-control study	24 patients, 75 controls	N/A	Blood	Neurologic parameters (Serotonin)	Diagnosis	67% of patients with tinnitus had increased blood serotonin levels compared to the reference value.

Abbreviations: TC: Total cholesterol; TRG: Triglyceride; LDL: Low-density lipoprotein; HDL: High-density lipoprotein; N/A: Not applicable; NLR: Absolute count of neutrophils/lymphocyte; PLR: Absolute count of platelet/lymphocyte; MPV: Mean platelet volume; HFHL-TN: High frequency-hearing loss-Tinnitus; AFHL-TN: All frequency-hearing-Tinnitus; NH-TN: Normal hearing-tinnitus; CNT: Control; BNDF: Brain-derived neurotrophic factor; SNP: Single nucleotide polymorphism; Val: Valine; Met: Methionine; ALP: Alkaline phosphatase; 11-dTxB2: 11-dehydro-thromboxane B2; TAS: Total antioxidant status; TOS: Total oxidants status; OSI: Oxidative stress index; AUCi: Area under the curve with respect to increase; TG: Triglyceride; THI: Tinnitus handicapped index; PTA: Pure tone audiometry; DPOAE: Distortion product otoacoustic emission; IL-2: Interleukin-2; CBT: Cognitive behavior therapy; TAC: Total antioxidant capacity; SOD: Superoxide dismutase; oxLDL: Oxidized LDL; IL-1α: Interleukin-1α; IL-10: Interleukin-10; TNF-α: Tumor necrosis factor-1α; HSP70: Heat shock protein-70; GDNF: Glial-derived neurotrophic factor; IMT: Intima-media thickness; VAS: Visual analogue scale; Cp: Oxidase ceruloplasmin; GSH: Glutathione; MDA: Malondialdehyde; cGPx: Glutathione peroxidase; GST: Glutathione S-transferase; SOD: Superoxide dismutase; BMI: Body mass index; LOOH: Lipid hydroperoxide; PC: Platelet count; PDW: Platelet distribution width; WBC: White blood cell; HPA; Hypothalamo-pituitary-adrenal axis; CCA: Common carotid artery; YEM: Young’s elastic modulus; CIMT: Common carotid intima-media thickness; SNHL: Sensorineural hearing loss; GRM7: Glutamate metabotropic receptor 7; NAT2: N-acetyltransferase 2; PON: Paraoxonase-1; TRT: Tinnitus retraining therapy; ESR: Erythrocyte sedimentation rate; CRP: C-reactive protein; sAA: Salivary α-amylase; IL-1β: Interleukin-1β, IL-6: Interleukin-6; NE: Norepinephrine; Epi: Epinephrine; 5-HIAA: 5-hydroxyindoleacetic acid; BDI: Beck’s depression inventory; BEPSI: Brief Encounter Psychosocial Instrument; DEX: Dexamethasone; HADS: Hospital anxiety and depression scale; 5-HTTLPR; 44-bp insertion-deletion in the promoter region; VNTR: 17-bp variable number tandem repeats; ACTH: Adrenocorticotropic hormone; 4-HNE: 4-hydroxynonenal.

## Data Availability

Not applicable.
